# Which phenotypic traits of resistance should be improved in cattle to control paratuberculosis dynamics in a dairy herd: a modelling approach

**DOI:** 10.1186/s13567-017-0468-8

**Published:** 2017-10-10

**Authors:** Racem Ben Romdhane, Gaël Beaunée, Guillaume Camanes, Raphaël Guatteo, Christine Fourichon, Pauline Ezanno

**Affiliations:** 1 BIOEPAR, INRA, ONIRIS, 44307 Nantes, France; 2 MAIAGE, INRA, 78352 Jouy-en-Josas, France

## Abstract

Paratuberculosis is a worldwide disease causing production losses in dairy cattle herds. Variability of cattle response to exposure to *Mycobacterium avium* subsp. *paratuberculosis* (Map) has been highlighted. Such individual variability could influence Map spread at larger scale. Cattle resistance to paratuberculosis has been shown to be heritable, suggesting genetic selection could enhance disease control. Our objective was to identify which phenotypic traits characterising the individual course of infection influence Map spread in a dairy cattle herd. We used a stochastic mechanistic model. Resistance consisted in the ability to prevent infection and the ability to cope with infection. We assessed the effect of varying (alone and combined) fourteen phenotypic traits characterising the infection course. We calculated four model outputs 25 years after Map introduction in a naïve herd: cumulative incidence, infection persistence, and prevalence of infected and affected animals. A cluster analysis identified influential phenotypes of cattle resistance. An ANOVA quantified the contribution of traits to model output variance. Four phenotypic traits strongly influenced Map spread: the decay in susceptibility with age (the most effective), the quantity of Map shed in faeces by high shedders, the incubation period duration, and the required infectious dose. Interactions contributed up to 12% of output variance, highlighting the expected added-value of improving several traits simultaneously. Combinations of the four most influential traits decreased incidence to less than one newly infected animal per year in most scenarios. Future genetic selection should aim at improving simultaneously the most influential traits to reduce Map spread in cattle populations.

**Electronic supplementary material:**

The online version of this article (doi:10.1186/s13567-017-0468-8) contains supplementary material, which is available to authorized users.

## Introduction

Bovine paratuberculosis or Johne’s disease (JD) is a bacterial infection caused by *Mycobacterium avium* subsp. *paratuberculosis* (Map). It mainly affects domestic ruminants. Paratuberculosis has a worldwide distribution with a high prevalence, herd prevalence being around 50% in Europe [1]. The progressive evolution of the infection leads to a chronic diarrhoea, an emaciation and death. This infection is responsible for significant weight losses, a decrease in milk production, an increase in mortality, and the early culling of infected animals, inducing economic losses [2]. Infectious animals shed bacteria in their faeces, milk, and colostrum. Susceptible animals are infected by ingesting Map or in utero. Calves are known to be the age group most susceptible to infection [3].

Individual response to a given exposure to Map differs among animals. Within-herd prevalence is usually low, with 2.8–27% of infected animals [4, 5]. Field observations have reported substantial variation in individual response to Map exposure: among birth cohorts which are assumed to have been similarly exposed to Map, some are later shown to be infected/infectious, while others remain not infected/infectious. In addition, the following observations have been made following experimental infection of similar aged calves with similar infectious dose of Map: (1) a wide range of paratuberculosis lesion severity have subsequently been observed [3], (2) different quantities of Map are shed in their faeces [6], and (3) different antibody responses have been detected, suggesting a variable duration of the latency period (being the period between infection and later detection by direct or indirect tests) [7]. The duration of the incubation period (which is defined as the period between infection and clinical signs) varied greatly between animals, ranging from 4 months to 15 years [8–12]. The amount of bacteria shed by infectious cattle is also highly variable, some being high shedders, while others are low shedders. Both intermittent and continuous shedding has been observed.

Individual resistance to paratuberculosis is assumed to be highly variable among, and expresses as different courses of infection. The phenotype of cattle resistance to paratuberculosis can be divided into (1) the ability to prevent infection and (2) the ability to cope with infection. This resistance in response to Map exposure involves different mechanisms and individual characters. Each of these characters will be denoted thereafter as phenotypic traits, a phenotype being defined by combined phenotypic traits. At the population scale, the distribution of phenotypic traits among individuals will influence the level of herd immunity, and therefore impair Map spread.

Strategies to control Map spread within dairy cattle herds usually consist in two main actions: hygiene improvement to reduce environmental and food contamination by Map, and a test-and-cull strategy to identify and remove infected animals. These control measures are not sufficient to control Map spread at herd and regional scales [13–15]. Vaccines against paratuberculosis have also been developed. Available vaccines decrease shedding of Map by infectious animals and decrease clinical signs of the disease [14, 16]. However, they do not prevent the infection of susceptible animals. In addition, most licensed vaccines show a cross reaction with tuberculosis diagnostic tests [17]. Therefore, the use of vaccination is restricted in many countries.

The observed variability of the individual response to Map exposure could support the development of innovative control measures applied at population scale if the most resistant animals can be selected. Several studies demonstrated a heritability of resistance to paratuberculosis in cattle ranging from 0.01 to 0.23 [17–21]. Recent studies highlighted an association between genetic markers and the course of Map infection [22–26]. Other genome markers were associated with Map shedding in faeces, presence of Map in several tissues, and seropositivity, in animals from comparable herds regarding paratuberculosis infection and of the same age group. Therefore, these animals were assumed to have been exposed in a similar way [25, 27, 28]. This highlights the potential to select for cattle more resistant to paratuberculosis. However, there are still gaps of knowledge concerning the phenotypic traits of resistance that would be the most relevant to improvements in the control of Map spread at population scale.

Modelling is the most appropriate approach to investigate the dynamics of complex systems such as within-herd Map transmission. Observational and experimental studies are both difficult to implement and expensive regarding the long evolution of paratuberculosis. In addition, a modelling approach allows us to overpass the lack of knowledge on genetic resistance of cattle to paratuberculosis by assuming improved phenotypic traits as if they were already selected for. Simulations then provide information on how such modifications of phenotypic traits would influence Map spread. Only one recent study investigated the potential effectiveness of hypothetical genetic selection as a strategy to control paratuberculosis at herd scale [29]. The authors assessed the effect of varying three phenotypic traits of resistance: (1) length of the susceptibility period, (2) level of the susceptibility to infection (expressed as the dose of Map required resulting in infection), and (3) duration of the latency period. Each tested phenotypic trait has been tested one-at-a-time and ranked by the time required to reach eradication. Modelling predictions showed that, when only genetic selection is implemented, eradication takes hundreds of years. However, this study did not investigate the potential progress in disease control when combining variations in several traits. In addition, other traits also could influence Map spread including intensity of shedding by infectious animals, in utero transmission, and progress of the infection course through different infection stages.

Our objective was to identify which phenotypic traits of resistance to paratuberculosis have the strongest influence on Map spread within a dairy cattle herd. The purpose was to identify ranges of phenotypic trait variations and trait combinations that limit Map spread in the herd. We assessed three categories of phenotypic traits both one-at-a-time and in combination, including: infection susceptibility, delays in the infection course, and shedding levels.

## Materials and methods

### Overall study design and model choice

A modelling approach was used to predict the effect of varying phenotypic traits of resistance to paratuberculosis on Map spread in a dairy cattle herd. We compared a situation where phenotypic traits were set at current observed levels with situations reached after a successful hypothetical genetic selection of more resistant animals in response to Map exposure. For each change of a trait, the resistance level was simulated as constant over time assuming that this average level had been reached in the population after a (not modelled) selection period. Several scenarios were simulated where one or several phenotypic traits were varied. The scenarios were compared regarding Map spread in the herd.

Several models have been published that represent Map spread within a dairy cattle herd (reviewed in [30], and more recently [29, 31–38]). We selected a stochastic compartmental model that offers an up-to-date description of Map spread within a dairy cattle herd. This model takes into account all of the major processes involved (according to the most recent literature) and allowed us to represent phenotypic traits of resistance corresponding to all of our hypotheses of interest. This model adequately combines demographical and infection dynamics, and accounts for herd structure, all these processes having been shown to highly influence Map spread [32]. The chosen model is mechanistic: each step and mechanism of the infection course is represented by a model parameter. This allowed us to simulate changes in phenotypic traits of resistance by minimal changes in the model.

### Main features of the model

The within-herd transmission model and the corresponding equations are fully described in Marcé et al. [32] and Beaunée et al. [15]. And a detailed description of the model is presented in Additional file 1.

**Figure 1:**
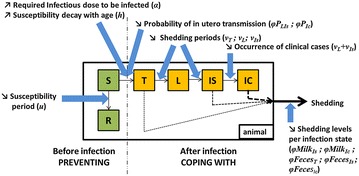
**Paratuberculosis infection course and phenotypic traits of interest to reduce Map spread at herd scale: in bracket: the corresponding parameters coding for them in the model.** Boxes, disease states; S, susceptible; R, no longer susceptible; T, transiently infectious; L, latent; Is, moderate shedder; Ic, high shedder or clinically affected animals. Green compartments: non infected states, orange compartments: infected states, dashed arrows: shedding, solid arrows: transitions between states, blue (large) arrows: changes in individual phenotypic traits that could limit Map spread at herd scale.

The main modelling assumptions are the following: the herd population dynamics reflects the one of a typical western Europe Holstein herd with 5 age groups (unweaned calves, weaned calves, young heifers, bred heifers, and cows), a high renewal rate of cows (one-third per year), and no males kept in the herd. The within-herd contact structure varies seasonally between housing and pasturing periods. The infection dynamics is represented by successive health states (Figure 1): animals initially susceptible (state *S*) are assumed to be no longer susceptible (state *R*) after a susceptibility period of duration *u*. Susceptibility decreases with age, assuming an exponential decay coefficient *h*. The possible infection of adults is neglected as it rarely occurs (it has only been demonstrated in adults following sudden exposure to a highly contaminated environment [39]). Infection can occur when a susceptible animal is in contact with a sufficient infectious dose per animal *α* (explicit indirect transmission), and then becomes transiently infectious (state *T*) for an average duration *ν*_*T*_. Then, infected animals enter a latent state (state *L),* during which shedding is neglected. After this latent period of average duration *ν*_*L*_, they become moderate shedders (state *Is*). For some animals, the evolution of the infection leads to a persistently high shedding and most of the animals are likely to have reduced milk yield or clinical signs called here high shedding and clinically affected state (*Ic*) after an average duration *ν*_*Is*_ in the moderate shedding state. Animals are assumed to be culled on average 6 months after entering *Ic* state.

Susceptible animals can be infected through five transmission routes [32]: (1) contact with bacteria present in the general environment of the farm contaminated by all shedders, (2) contact with bacteria present in the local environment of calves contaminated by shedding calves, (3) in utero transmission from infected cows to their foetus, and (4) ingestion of contaminated milk or (5) colostrum from infectious cows.

### Phenotypic traits of resistance to paratuberculosis

In this study we assessed the effect of varying 14 phenotypic traits of resistances to paratuberculosis and combination of them on Map transmission in the herd. Each of the tested scenarios corresponds to a variation, or combinations in variation, in one or more phenotypic trait of resistance to paratuberculosis.

The phenotype of cattle resistance to paratuberculosis is classically divided into (1) resistance to infection defined as the ability to prevent infection when exposed to a given dose of Map, and (2) tolerance to infection defined as the ability to cope with infection when infected [40, 41]. On the one hand, animals are considered to be resistant (ability to prevent infection) if they show a decrease in susceptibility to infection if they are no longer susceptible at a younger age, if they need to be exposed to a higher dose of Map to be infected, or if they show a faster decrease in susceptibility with age than less resistant animals. On the other hand, animals are considered tolerant (ability to cope with infection once infected) if they show longer latency and incubation periods, and a lower shedding level when infectious than less tolerant animals. In addition, foetuses of the latter may have a lower chance to be infected in utero.

We accounted for all of the potential mechanisms involved in an increased resistance to paratuberculosis because we assumed they can all contribute to Map spread at herd scale (Figure 1). The ability to remain non-infected was composed of four components: (1) a shorter susceptibility period for calves, (2) a faster decrease in age-related susceptibility, and (3) a higher infectious dose of Map needed to be infected after birth. The ability to cope with infection was represented by a longer latency period before the onset of moderate shedding, a longer incubation period before high shedding and clinical signs, a decrease in the amount of Map shed through the different transmission routes and a decrease in the probability of in utero transmission. Overall, we studied 14 parameters coding for the identified phenotypic traits of resistance to paratuberculosis (Table 1).

**Table 1:** **Parameters coding for the phenotypic traits of resistance: definition and values**

Parameters	Definition	Reference value	Univariate simulations: [min–max]		Multivariate simulations: tested values				Source
					#1	#2	#3	#4	
*u*	Susceptibility period duration	52 weeks	[1–52]		–				[51–53]
*h*	Decay in susceptibility with age (coefficient)	0.1	[0.1–1]		0.2	0.3	0.4	0.5	[54]
*α*	Required infectious dose to be infected	10^6^ bacteria	[10^6^–10^12^]		1.5 × 10^6^	2 × 10^6^	2.5 × 10^6^	3 × 10^6^	[55]
*ν*_*L*_	Duration of latent state	52 weeks	[52–208]		–				[10, 11]
*ν*_*L*_ *+* *ν*_*Is*_	Duration before high shedding and clinically affected state	156 weeks	[156–468]		234	312	390	468	[10–12]
*v*_*T*_ *with ν*_*T*_ *+* *ν*_*L*_ = constant	Duration of transiently infectious state with constant duration before moderate shedding state	νT = 25 weeks (νT + νL = 77)	[1–25]		–				[8–11]
*ν*_*Is*_ *with ν*_*L*_ *+* *ν*_*Is*_ = constant	Duration of moderate shedding state with constant duration before high shedding or clinically affected state	νIs = 104 weeks (νL + νIs = 156 weeks )	[60–104]		95	86	77	68	[8–12]
*φMilk*_*X*_	Factor of decrease of Map shed in milk by animals in health state X								
*φMilk*_*Is*_	Moderate shedding state (*Is*)	100%	[0–100]		50%	10%	5%	0%	[56]
*φMilk*_*Ic*_	High shedding and clinically affected state (Ic)	100%	[0–100]						[57]
*φFeces*_*X*_	Factor of decrease of Map shed in faeces by animals in health state X								
*φFeces*_*T*_	Transient state (*T*)	100%	[0–100]		50%	10%	5%	0%	[9]
*φFeces*_*Is*_	Moderate shedding state (*Is*)	100%	[0–100]						[58]
*φFeces*_*Ic*_	High shedding or clinically affected state (*Ic*)	100%	[0–100]		66%	50%	40%	33%	[59, 60]
*φP*_*X*_	Factor of decrease of probability of in utero transmission for cows in health state X								[2, 61]
*φP*_*LIs*_	Latent and moderate shedding states (*LIs*)	100%	[0–100]		50%	10%	5%	0%	
*φP*_*Ic*_	High shedding or clinically affected state (*Ic*)	100%	[0–100]						

Based on the literature, we defined a realistic variation of resistance levels to simulate within observed values for the investigated traits. The reference value was the worst one. Changes were simulated from reference to the most favourable value observed value, Indeed, calves susceptibility can sharply reduce, and animals are no longer susceptible, as soon as their first week of life [53–56]. Some susceptible animals have been shown to need a dose of bacteria as high as 10^12^ to become infected [57]. After a transient shedding period, infected animals can have a barely detectable level of shedding for about 4 years (208 weeks) [10, 11]. Infected animals can show clinical signs of the disease up to more than 9 years after infection (468 weeks). Concerning the probability of transmission of Map in utero from infected dam to its foetus and the quantities of bacteria shed through different routes, only partial information was available. Hence, we chose to test for extreme values by assuming that animals can stop shedding completely with no further in utero transmission of the infection. Nevertheless, it has been shown that high shedders and clinically affected animals can shed as few as 10^8^ bacteria/kg of faeces, which corresponds to 1/100^th^ of the reference value that we have assumed in our model [58, 59].

### Initial conditions and model outputs

Map spread was initiated by the introduction of a moderate shedding cow into a fully naïve herd of 260 animals. We assumed that herd renewal is mainly driven by internal demographic processes (no further introduction), which is typical of western Europe farming systems. Map spread was predicted over 25 years. To obtain accurate outputs from the stochastic model, we ran 500 repetitions for each of the tested scenario. A scenario represented one phenotype of interest. Each phenotype was defined by a set of values of 14 parameters.

Four model outputs described Map spread within a herd (Table 2). All outputs were calculated at the end of the simulation, *t* = 25 years after Map introduction. The first output was the cumulative incidence calculated as the mean cumulative number of newly infected animals over the 25 years of simulation. The second output was the infection persistence defined as the proportion of runs where the infection persisted until 25 years after Map introduction, i.e. where there was at least one infected animal of state *T, L, Is*, or *Ic*, or bacteria in the environment. The third output was the prevalence of infected animals calculated as the median prevalence of infected animals in the population 25 years after Map introduction for runs where the infection persisted. Finally, the fourth output was the prevalence of affected animals calculated as the median prevalence of high shedding and clinically affected animals in the population 25 years after Map introduction for runs where the infection persisted. Outputs related to prevalence were calculated only if Map persistence was higher than 6% (30 runs out of 500) in order to provide a sufficient number of runs to estimate medians.

**Table 2:** **Contribution of the four most influential phenotypic traits to the model output variance**

	Parameters	Cumulative incidence	Persistence	Prevalence of infected animals	Prevalence of affected animals
Principal effect	h	*0.25*	*0.34*	*0.30*	*0.22*
	νL + νIs	*0.16*	*0.16*	*0.18*	*0.22*
	φFecesIc	*0.14*	*0.17*	*0.14*	*0.09*
	α	*0.13*	*0.17*	*0.12*	*0.08*
First order	h:νL + νIs	*0.07*	*0.05*	*0.09*	*0.12*
	h:φFecesIc	*0.06*	0.04	*0.06*	0.03
	h:α	*0.06*	0.04	*0.05*	0.03
	νL + νIs:φFecesIc	0.03	0.02	0.03	0.04
	α:νL + νIs	0.03	0.02	0.03	0.03
Second order	h:α:νL + νIs	0.03	2 × 10^−3^	0.02	0.03
	h:νL + νIs:φFecesIc	0.03	10^−3^	0.02	0.03
	α:φFecesIc	0.02	0.02	0.01	6 × 10^−3^
	h:α:φFecesIc	0.02	2 × 10^−4^	5 × 10^−3^	10^−3^
	α:νL + νIs:φFecesIc	8 × 10^−3^	3 × 10^−4^	2 × 10^−3^	4 × 10^−3^
Third order	h:α:νL + νIs:φFecesIc	3 × 10^−3^	3 × 10^−3^	4 × 10^−4^	7 × 10^−5^
	Residuals	0.31	0.15	0.27	0.38

Contribution was estimated from the ANOVA. In italic, values above 5%.

### Simulation protocol and output analysis

First, we performed a univariate simulation study: each of the traits of interest was varied one-at-a-time, assuming they varied independently (Table 1). Second, we performed a multivariate simulation study: combinations of phenotypic traits were studied to test for a potential enhanced effect of simultaneously improving several phenotypic traits simultaneously. The R programming language [42] was used for data analyses. Results obtained in the univariate simulation study revealed that some parameters—when analysed one-at-a-time—did not influence model outputs. Instead of keeping numerous parameters or removing some of them expected not to be influential, we grouped in this second step non-influential parameters when they are untangled in the same trait or when involved in a given transmission route. This decrease in the number of considered parameters without losing information eased the interpretation of the multivariate simulation study results. Ranges of variation of phenotypic traits were represented by five possible values per trait (including the reference value) combined in the multivariate simulation study using a complete factorial design, leading to 390 625 scenarios (Table 1). Five levels of variation per trait appeared to be a good compromise between parameter space exploration and number of scenarios to investigate interactions. A complete factorial design was required to assess all interaction orders.

We performed a cluster analysis of the multivariate scenarios based on two of our model outputs. Scenarios were grouped to minimise outputs variability within a cluster and maximise this variability among clusters. The aim was to identify and characterize groups of scenarios. We build clusters using the two model outputs available on all model repetitions of each scenario: cumulative incidence and infection persistence 25 years after Map introduction, after they were standardized into variables of comparable scales. To define the appropriate number of clusters, we studied the sum of squared distances between each scenario and the centroid of its corresponding cluster (called the sum of squared error or the within-group sum-of-squares) for different number of clusters [43–45]. Clusters were built using k-means clustering method (*kmeans* function from R package “*FactoMineR*” [46]). A descriptive analysis was performed to characterize clusters for phenotypic traits using *catdes* function (“*FactoMineR*” package [46]). This step aimed to identify if tested variations in phenotypic traits are uniformly distributed in regards of cluster or if some values are over represented in a given cluster. Besides, we performed an ANOVA to quantify the contribution of each trait to the variance of each of the four model outputs. Each trait contribution to the model output variance (*κ*) was calculated as:

$$ \begin{aligned} \kappa & = principal\;effect\;of\;the\;trait \\ & \quad + \sum\nolimits_{i = 1}^{i = m} {\frac{ith\;order\;interaction\;effect\; involving\;the\;trait}{i + 1},} \\ \end{aligned} $$

with *i* the interaction order and *m* the highest interaction order in which the trait was involved. A second ANOVA was performed on the influential phenotypic traits to quantify contribution of each trait (principal effect) and each combination of them (interaction) to the variance of each of the four model outputs. Factors (individual traits or combinations of traits) were influential if they contributed to more than 5% of the variance of at least one of the four model outputs.

In order to identify the most effective combinations of variation in phenotypic traits to decrease Map spread, we used the cumulative incidence output as an indicator of a successful Map control at herd scale. For each combination, the cumulative incidence was plotted and visually described (Figure 6). In addition, we chose two thresholds to evidence the most effective combinations with emphasis on the ones with the lowest variations in parameters (Figures 6, 7): (1) 25 newly infected animals over the 25 years of simulation, interpreting such a level of one newly infected animal per year as an infection under control, and (2) half this threshold, i.e. 12 newly infected animals over the 25 years of simulation.

## Results

**Figure 2:**
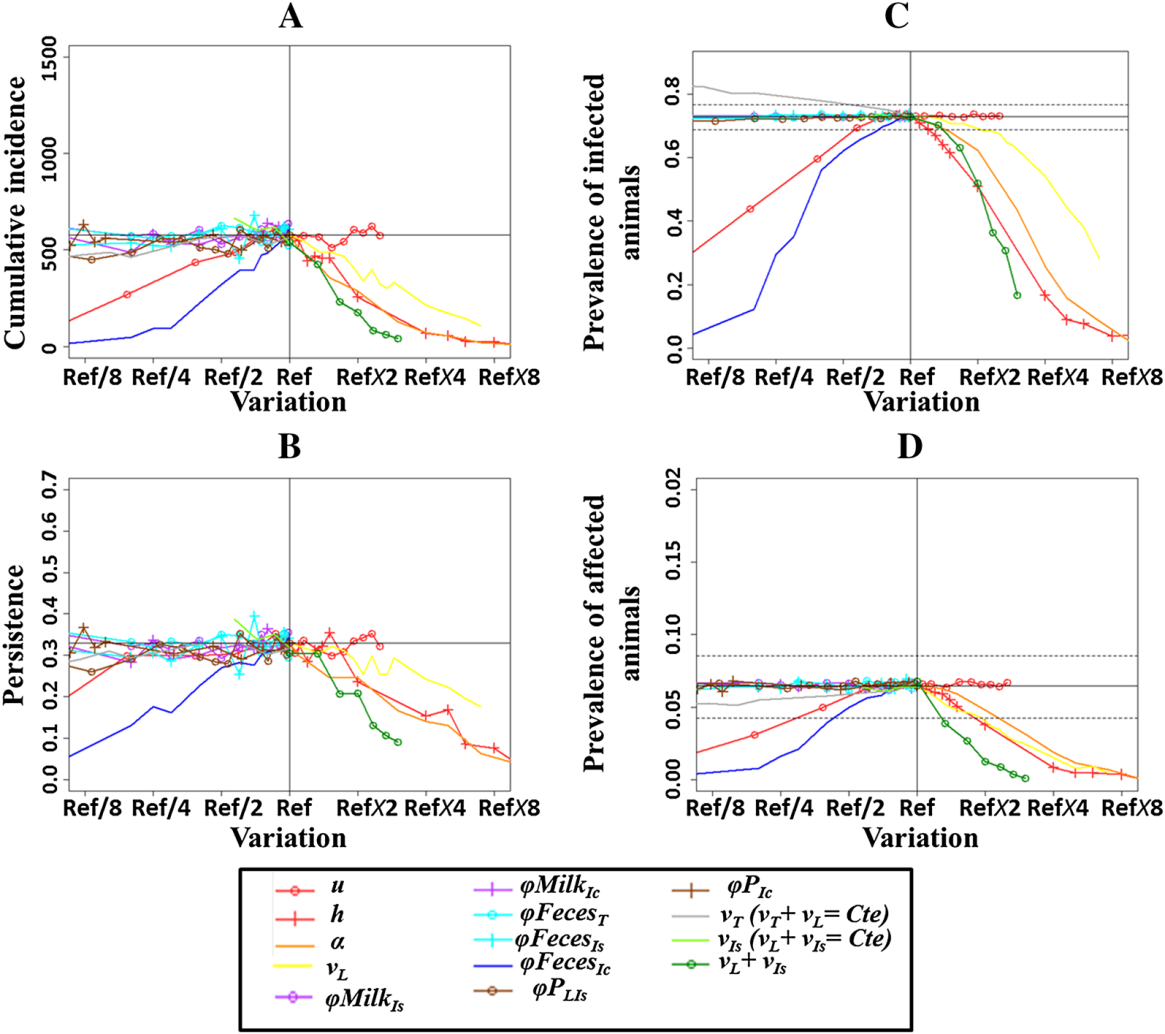
**Changes in model outputs resulting from univariate variations of phenotypic traits of resistance to paratuberculosis. A** cumulative incidence, **B** persistence, **C** prevalence of infected animals, **D** prevalence of high shedders and affected animals at the end of simulations (25 years). “Ref” corresponding to the reference value of the phenotypic trait. See Table 1 for parameter definitions and tested values, Table 2 for output definitions. Vertical and horizontal solid lines give reference values, dotted lines give associated 5^th^ and 95^th^ percentiles.

In the univariate simulation study the variation of six phenotypic traits influenced at least one model output resulting in decrease in Map spread (Figure 2): a shorter susceptibility period (*u*), an increase in the decay in susceptibility with age (*h*), an increase in the required infectious dose (*α*), a longer latent state (*v*_*L*_), a delayed occurrence of the high shedding or clinically affected state (*v*_*L*_ + *v*_*Is*_), and a decrease in the quantity of Map shed in faeces by high shedders or clinically affected animals (*φFeces*_*Ic*_).

Eight of the traits investigated in the univariate simulation study did not influence Map spread dynamics. These traits were: a shorter transiently infectious state when assuming a constant duration before the moderate shedding state (*v*_*T*_ with *v*_*T*_ + *v*_*L*_ = *constant*), a shorter latent state when assuming a constant duration before the high shedding and clinically affected state (*v*_*Is*_ with *v*_*L*_ + *v*_*Is*_ = *constant*), a decrease in the quantity of Map shed in milk by moderate shedders (*φMilk*_*Is*_) and high shedders and clinically affected animals (*φMilk*_*Ic*_), a decrease in the quantity of Map shed by transiently infectious animals (*φFeces*_*T*_), and by moderate shedders (*φFeces*_*Is*_), and a decrease in the probability of in utero transmission by latent infectious animals and moderate shedders (*φP*_*LIs*_), and by high shedders and clinically affected animals (*φP*_*Ic*_).

We chose traits to be included in the multivariate simulation study in light of these results, noting that it was not possible to evaluate interactions among traits could have been evaluated with such a univariate analysis. Among traits highlighted as influential, we kept all except *u* that was redundant with *h*. Among other traits, we kept *v*_*Is*_ (assuming *v*_*L*_ + *v*_*Is*_ = *constant*) and we grouped traits related to Map shedding in milk and colostrum (*φMilk*), to in utero transmission (*φP*), and to Map shedding in faeces by transiently infectious animals and moderate shedders (*φFeces*_*TIs*_).

**Figure 3:**
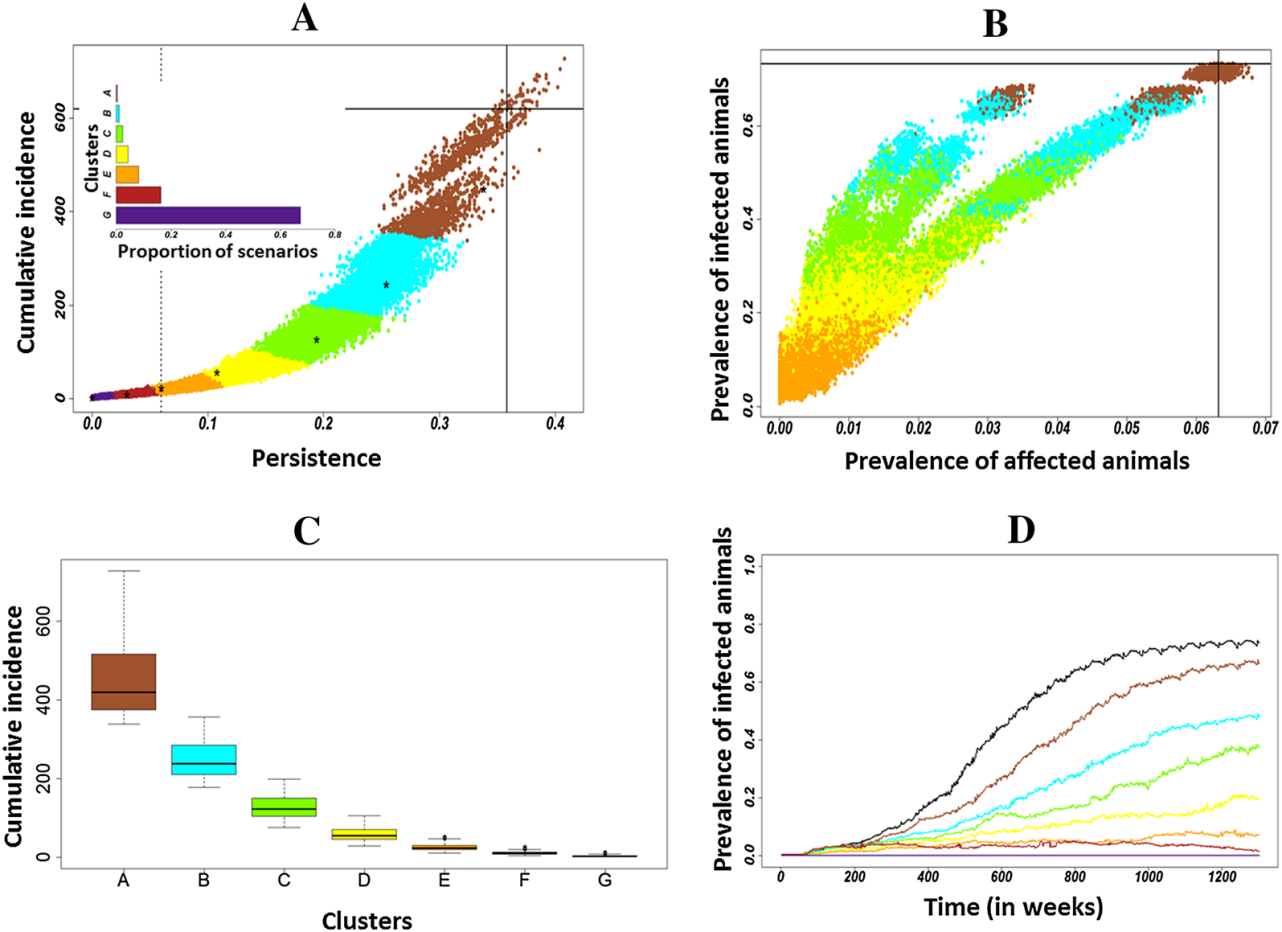
**Model outputs 25** **years after initial Map introduction for all of the multivariate scenarios: scenarios were clustered by cumulative incidence and persistence. A** cumulative incidence and persistence per scenario and proportion of scenarios per cluster; **B** prevalence of infected animals and of affected animals per scenario where persistence was higher than 6%; **C** boxplots of the cumulative incidence for each cluster; **D** evolution over time of the prevalence of infected animals for the centroids of the seven clusters (A–G). Solid lines show output reference values, the dashed line represents the threshold of 30 runs where infection persists, asterisks indicate centroids of clusters. Total number of scenarios is 380 625.

The cluster analysis of multivariate scenarios identified seven groups of scenarios from current (A; assumed as the worst) to the best control of Map spread (G; Figures 3A and B). This analysis highlighted three distinct dynamics (Figure 3): clusters A and B represented low control with a decrease in cumulative incidence, a slight decrease in infection persistence, and an almost as high prevalence of infected animals. Clusters C, D, and E represented a good control with a low cumulative incidence, persistence and prevalence of infected animals, but with the occurrence of high shedders and clinically affected animals. Clusters F and G represented complete control with a very low cumulative incidence and persistence. Up to 80% of the scenarios were in these most favourable clusters F and G (Figure 3A).

**Figure 4:**
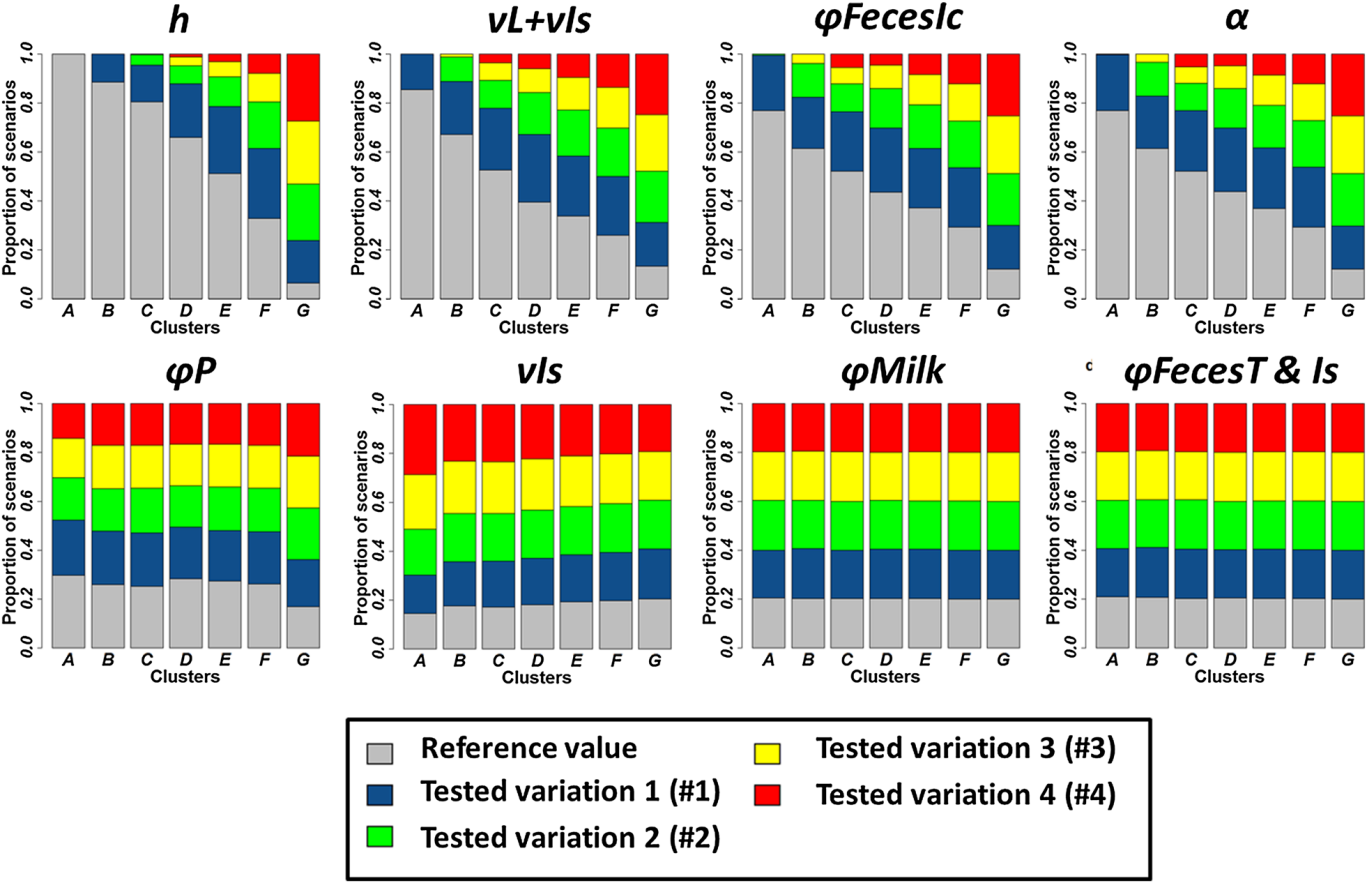
**Distribution of scenarios among tested values for each phenotypic trait per cluster (A–G).** See Table 1 for parameter definitions and values, and Figure 3 for cluster definition.

**Figure 5:**
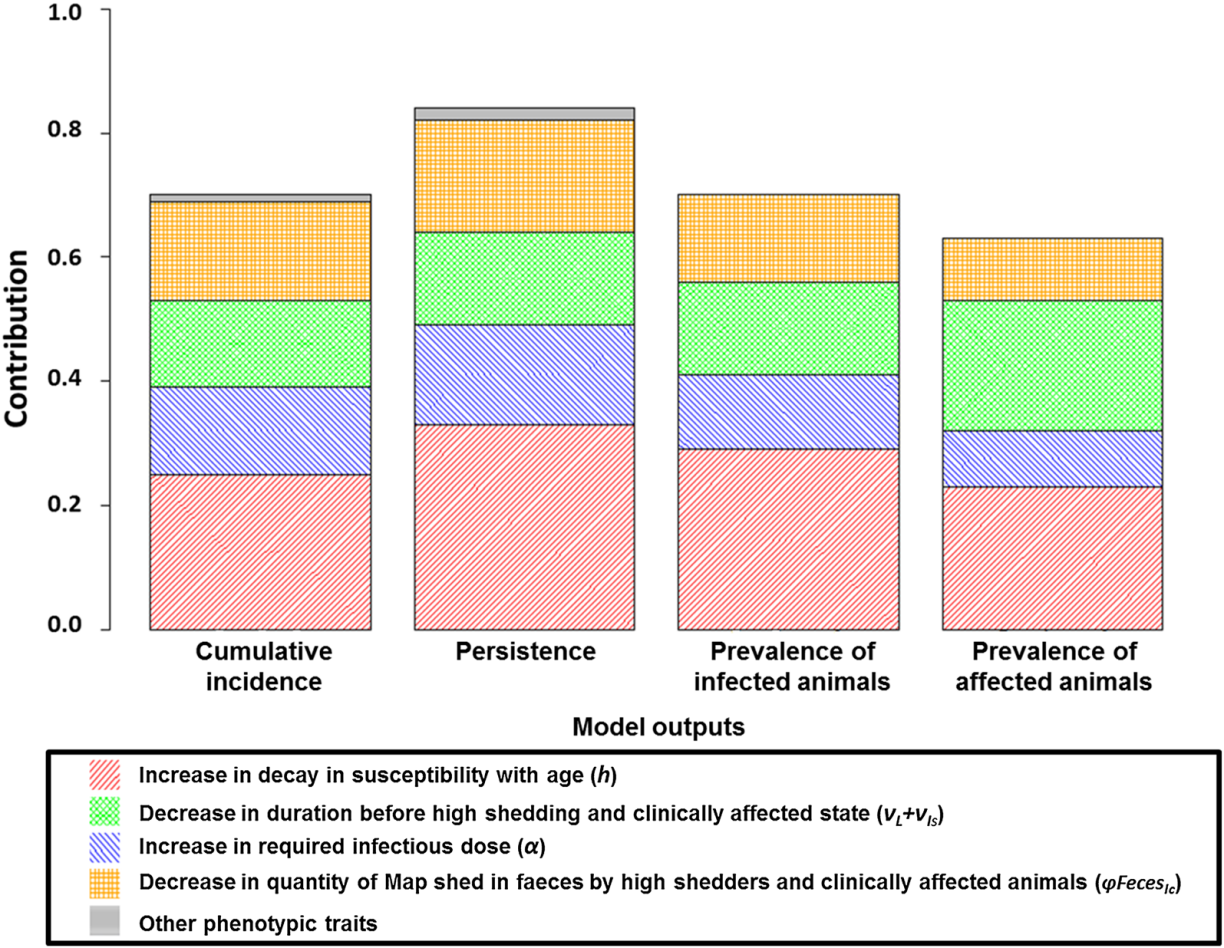
**Total contribution of phenotypic traits to model output variance.** Contribution includes the principal effect of a given factor and interaction effects in which this factor was involved divided by the number of factors involved.

The descriptive analysis (Figure 4) of clusters showed that the dynamics toward the most favourable clusters was mainly driven by four out of the eight traits: increasing the decay in susceptibility with age (*h*), lengthening the incubation period (*ν*_*L*_ + *ν*_*Is*_), decreasing the quantity of Map shed in faeces by high shedders or clinically affected animals (*φFeces*_*Ic*_), and increasing the required infectious dose (*α*). The ANOVA (Figure 5) evidenced that these four phenotypic traits contributed most to the variance of model outputs, and allowed us to rank phenotypic traits from the most to the less influential. The increase in the decay in susceptibility with age (*h*) contributed the most to the variance of the four model outputs, while a decrease in the quantity of Map shed in faeces by high shedders and clinically affected animals (*φFeces*_*Ic*_), a longer incubation period (*ν*_*L*_ + *ν*_*Is*_), and an increase in the required infectious dose (*α*) led to almost equivalent contributions to model output variances.

Combined variations of phenotypic traits of resistance contributed to decrease Map spread dynamics. Interactions among traits showed contributions to model output variance ranging from 0.007% to up to 12% (Table 2). The interaction between increased decay in susceptibility with age (*h*) and a lengthened incubation period (*ν*_*L*_ + *ν*_*Is*_) contributed to 12% of the variance of the prevalence of affected animals, and was also the most contributing interaction for other model outputs. In addition, *h* was involved in all of the contributing interactions, thus having both the highest principal and interaction effects.

**Figure 6:**
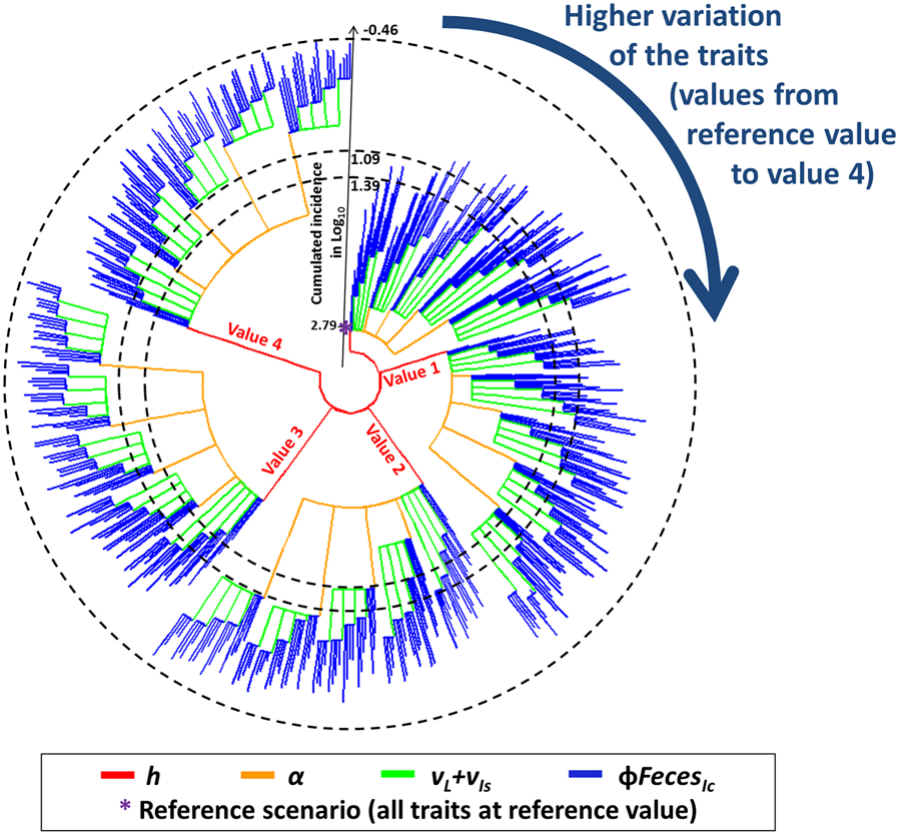
**Effect of combined variations of the four most influential phenotypic traits on cumulative incidence.** Cumulative incidence was calculated 25 years after initial Map introduction. The external dashed circle corresponds to the lowest cumulative incidence (0.35 newly infected animals) obtained among tested scenarios (log_10_(0.35) = − 0.46), the internal dashed circles correspond to thresholds of 25 (log_10_(25) = 1.39) and 12 (log_10_(12) = 1.09) newly infected animals. Asterisk corresponds to the cumulative incidence (log_10_(617) = 2.79) for the scenario with current values of phenotypic traits. *h*, decay in susceptibility with age; *α*, increased required infectious dose; *ν*_*L*_ + *ν*_*Is*_, increased duration before high shedding or clinically affected state; *ϕFeces*_*Ic*_, decreased quantity of bacteria shed by high shedders or clinically affected animals. Each leaf is one scenario with branches representing variations of the four traits. Scenarios are presented by increasing level of variation in each trait successively (*h*, *α*, *ν*_*L*_ + *ν*_*Is*_, and, *ϕFeces*_*I*_). Tested values are given in Table 1.

**Figure 7:**
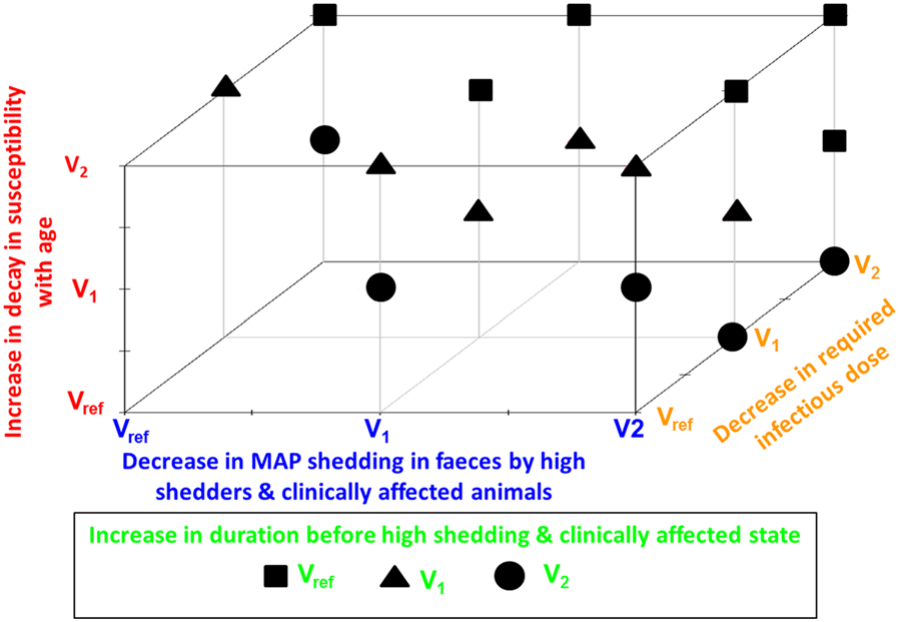
**Threshold of variation in influential parameters and combinations necessary to reach a low cumulative incidence.** Squares, triangles and dots represent the threshold value for the parameter “duration before high shedding and clinically affected state” needed to reach the cumulative incidence <25 over 25 years of simulation at the given value of the three other influential parameters (required infectious dose, Map shedding in faeces by high shedders and clinically affected animals, and decay in susceptibility). The ten empty positions corresponds to combinations in variations where “duration before high shedding and clinically affected state” have to be more than doubled (>V2) to have a low cumulative incidence (<25). Combinations represented here account only for threshold of variations below V2. Tested values (Vref, V1, and V2) are given in Table 1.

The combined variation in the four most influential phenotypic traits of resistance to paratuberculosis decreased the collative incidence to <1 newly infected animal over 25 years of simulation when set at their highest tested level. Over the 625 scenarios combining variations of the four most influential phenotypic traits, 537 scenarios resulted in decrease the cumulative incidence from 617 newly infected animals when phenotypic traits were set to their current values to 25 newly infected animals over the 25 years of simulation, and 473 scenarios allowed a cumulative incidence to be reached of 12 newly infected animals over the 25 years of simulation (Figure 6). Fourteen of the tested scenarios allowed to achieve a good control of the disease dynamics in the herd (<25 newly infected animals over the 25 years of simulation) with one of the four most influential traits at its reference value and the other trait at value 1 or value 2 (Figure 7). Some of the tested scenarios allowed to decrease the cumulative incidence to 25 newly infected animals over the 25 years of simulation or less were based on moderate variations of phenotypic traits (Figure 7). For example, a combined variation of the four most influential traits at their first tested level (#1) led to a cumulative incidence of 19 newly infected animals. On the other hand, improving a single trait, even a fivefold increase in the decay in susceptibility with age (#4), the most influential trait, was not sufficient to reach accumulative incidence of 25 newly infected animals or less over the 25 years of simulations. Interesting examples of combined moderate variations of traits allowing decreasing the cumulative incidence were highlighted. First, halving the decay in susceptibility with age (*h*) (#2) together with a 50% increase in duration before entering the high shedding and clinically affected state (*V*_*L*_ + *V*_*Is*_) (#1), and a 34% decrease in the quantity of bacteria shed by high shedders or clinically affected animals (*ϕFecesIc*) (#1) results in threshold being reached of 23 newly infected animals over the 25 years of simulation. Second, tripled threefold increase in the decay in susceptibility with age (*h*) (#2) combined with a doubling of the required infectious dose (*α*) (#2), and a 34% decrease in the quantity of bacteria shed by high shedders or clinically affected animals (*ϕFeces*_*Ic*_) (#1) resulted in a cumulative incidence of 13 newly infected animals.

## Discussion

Variations of 4 of 14 phenotypic traits strongly reduced Map spread within a dairy cattle herd: the decay in susceptibility with age, this being the most influential trait, the quantity of Map shed in faeces by high shedders and clinically affected animals, the duration of the incubation period, and the required infectious dose. Combining these phenotypic traits was the sole way to effectively control Map spread at the herd scale. Most tested combinations of these influential phenotypic traits allowed the cumulative incidence to be reduced to <25 newly infected animals over the 25 years of simulation, which was interpreted here as an infection under control. Interestingly, such a low level of cumulative incidence could not be reached when varying a single phenotypic trait.

The increase in the decay in susceptibility with age is largely related to a shorter susceptibility period. We also highlighted the required infectious dose as an influential phenotypic trait. Our results concerning these traits are in agreement with van Hulzen et al. [29], who in a theoretical study also identified that an earlier resistance acquisition would be crucial when it comes to control paratuberculosis using genetic selection. However, there is nowadays no available knowledge to implement a genetic selection on these traits. These traits are not easily measurable in field conditions.

A decrease in the quantity of Map shed in faeces by high shedders and clinically affected animals, which was also identified as an influential phenotypic trait, might be achieved thanks to genetic selection. Currently, it has been shown that genetic markers could be associated with the occurrence of shedding versus no shedding at all by animals in infected herds [23, 27, 47]. More precise knowledge is needed concerning our ability to select cattle that will shed less Map in faeces while in their last stage of infection.

While van Hulzen et al. [28] identified the increase in duration of the latency period as an effective phenotypic trait in controlling paratuberculosis through genetic selection, we highlighted that an increase in this latency period (this being the period between infection and the occurrence of a moderate detectable shedding) without delaying the start of the high shedding or clinically affected state did not influence Map spread dynamics in the herd. We have shown that it will be more interesting to lengthen the incubation period, as this delays the occurrence of the high shedding or clinically affected state.

Phenotypic traits identified as influencing Map spread dynamics at the herd scale also are related to control measures currently implemented in infected herds in the field [14]. Therefore, a valuable interaction can be expected between routine control plans and innovative control through genetic selection.

The variation of several other traits did not influence Map spread dynamics: decrease in duration of transiently infectious state with a constant duration before moderate shedding state, decrease in quantity of Map shed in milk and colostrum irrespective of the animal infection state, decrease in quantity of Map shed in faeces by transiently infectious animals and moderate shedders, and decrease in probability of in utero transmission irrespective of animal infection state. A decrease in duration of moderate shedding state (from 104 to 60 weeks) did not influence Map spread dynamics. The range of variation modelled for this trait was lower than for other traits due to limitations inherent to the compartmental model. Nevertheless, as no effect was evidenced with a reduction of one-third of that duration, we assumed that this trait was not highly influential over the simulated range.

The four traits identified as influential are well described in the literature therefore we can assume that their tested ranges of variation were realistic. We assumed extreme ranges of variation for traits for which information was missing. The other traits assessed were not influential even with such extreme, non-realistic, variations. Using a different set of variation in the investigated traits is not expected to change our conclusions concerning which traits influence Map spread within dairy cattle herds.

As our objective was to assess Map spread in herds in which phenotypic traits would have been improved, we did not account for the long time needed [29] to reach such targeted levels of phenotypic traits by a potential genetic selection. On the one hand, recent studies identified several genetic markers associated with resistance (reviewed in [48, 49]), but genes and mechanisms responsible for the tested phenotypic traits are still unknown. Further genetic studies of resistance of cattle to paratuberculosis are required, especially to identify genes and mechanisms involved in these relevant phenotypic traits to allow potential future selection of more resistant cattle. In addition, diagnostic tests currently available in the field do not allow identifying animals having the phenotypic traits identified here. Concerning future genetic selection, tests more sensitive during the early stage of the infection would be needed to distinguish infected animals from others and to better quantify the individual duration of incubation periods. On the other hand, there is a risk of a negative association between phenotypic traits of resistance to paratuberculosis and other traits of economic importance. For example, it has been shown that genetic markers associated with susceptibility to paratuberculosis could be associated to lactation persistence [50].

Our model represents a typical western Europe farming system for dairy cattle herds. Demographic processes have been shown to highly influence the disease dynamics [32], and therefore, different farming systems could change the influence of the studied phenotypic traits on Map spread dynamics in the herd. We assumed a closed herd without introduction of animals from other herds. Animal exchanges between herds could reintroduce Map in free herds and thus influence disease dynamics. However, it is not expected to modify our conclusions as regards the identification of crucial phenotypic traits to better manage infected herds. Indeed, a single Map introduction can lead to infection persistence in 40% of the cases under current situation as regards phenotypic traits [32], with a huge cumulative incidence reached after 25 years if no control is applied. Animal movements are not expected to modify significantly this finding. However, the occurrence of animal movements might increase the cumulative incidence under controlled situations with improved traits.

This study highlighted four phenotypic traits of resistance of cattle to paratuberculosis influencing Map spread within a dairy herd: decay in susceptibility with age, quantity of Map shed in faeces by high shedders and clinically affected animals, duration of the incubation period, and required infectious dose. A combination of these traits strongly contributes to limit Map spread. Further genetic study should aim at better identifying cattle genes involved in these traits in order to allow their potential selection.

## Additional file

**Additional file 1.** **Description of the used within herd model of Map spread.**
