# Supplementary material for: Which phenotypic traits of resistance should be improved in cattle to control paratuberculosis dynamics in a dairy herd: a modelling approach
Source: Vet Res. 2017 Oct 10;48:62. doi: 10.1186/s13567-017-0468-8 (PMC5634854; doi:10.1186/s13567-017-0468-8)
Supplement: Supplementary file 1 — Additional file 1. Description of the used within herd model of Map spread. [file 13567_2017_468_MOESM1_ESM.pdf]

Additional file 1: Description of the used within herd model of Map Spread

A Equations for the within-herd dynamic

The description of the model we used corresponds to the within-herd model described in [2] and based on [11]. The set of parameters used are described in tables 1, 2 and 3.

Notations

In the following equations, $X_{(t,a)}$ is the number of animals in health state X at time t and age a. Age is given in weeks until the entry in the adult stage (from 1 to cal, with intermediary stages w for weaning age, y for young heifers age, h for heifers age and u for maximal age in the susceptible compartments), then by age group (P_1 to P_{5+}). Some variables can have a prefix: "b" for births in health states X (bX), "N" for animals transiting between two health states ($\text{N}X$) and "s" for exits (mortality and culling) (sX). After entering the adult stage, flows corresponding to aging are noted using a superscript ng, whereas those remaining in the same age group are noted using a superscript sg. $N_{(t,a)}$ is the number of animals of age a at time t. Average duration in health states are noted by v_X. The remaining terms used are defined when introduced.

Equations for the updating of variables describing health states

In this section we introduce the equations for the updating of variables corresponding to the health states, for a given herd i.

Susceptible (S) and No more Susceptible (R)

$$S_{(t+1,a=1)} = bS_{(t)} - \text{N}T_{(t+1,a)}$$

$$S_{(t+1,a\in[2;52])} = \left[S_{(t,a-1)} - sS_{(t,a-1)}\right] - \text{N}T_{(t+1,a)}$$

$$R_{(t+1,53)} = S_{(t,52)} - sS_{(t,52)}$$

$$R_{(t+1,a\in[54;cal])} = R_{(t,a-1)} - sR_{(t,a-1)}$$

$$-$$

$$R_{(t+1,P_1)} = R^{sg}_{(t,P_1)} - sR^{sg}_{(t,P_1)} + R_{(t,cal)} - sR_{(t,cal)}$$

$$R_{(t+1,P_i\in[P_2;P_4])} = R^{sg}_{(t,P_i)} - sR^{sg}_{(t,P_i)} + R^{ng}_{(t,P_{i-1})} - sR^{ng}_{(t,P_{i-1})}$$

$$R_{(t+1,P_{5+})} = R_{(t,P_{5+})} - sR_{(t,P_{5+})} + R^{ng}_{(t,P_4)} - sR^{ng}_{(t,P_4)}$$

Transiently infected (T)

$$T_{(t+1,a=1)} = \ bT_{(t)} + \text{N}T_{(t+1,a)}$$

$$T_{(t+1,a\in[2;52])} = \left[T_{(t,a-1)} - sT_{(t,a-1)}\right] - \text{N}L_{(t+1,a)} + \text{N}T_{(t+1,a)}$$

$$T_{(t+1,a\in[53;cal])} = \left[T_{(t,a-1)} - sT_{(t,a-1)}\right] - \text{N}L_{(t+1,a)}$$

Latently infected (L)

$$L_{(t+1,a=2)} = \ \text{N}L_{(t+1,a)}$$

$$L_{(t+1,a\in[3;h])} = \ \left[L_{(t,a-1)} - sL_{(t,a-1)}\right] + \text{N}L_{(t+1,a)}$$

$$L_{(t+1,a\in[h+1;cal])} = \ \left[L_{(t,a-1)} - sL_{(t,a-1)}\right] - \text{N}Is_{(t+1,a)} + \text{N}L_{(t+1,a)}$$

$-$

$$L_{(t+1,P_1)} = \ \left[L^{sg}_{(t,P_1)} - sL^{sg}_{(t,P_1)} + L_{(t,cal)} - sL_{(t,cal)} + T_{(t,cal)} - sT_{(t,cal)}\right] - \text{N}Is_{(t+1,P_1)}$$

$$L_{(t+1,P_i\in[P_2;P_4])} = \left[L^{sg}_{(t,P_i)} - sL^{sg}_{(t,P_i)} + L^{ng}_{(t,P_{i-1})} - sL^{ng}_{(t,P_{i-1})}\right] - \text{N}Is_{(t+1,P_i)}$$

$$L_{(t+1,P_{5+})} = \left[L_{(t,P_{5+})} - sL_{(t,P_{5+})} + L^{ng}_{(t,P_4)} - sL^{ng}_{(t,P_4)}\right] - \text{N}Is_{(t+1,P_{5+})}$$

Moderate shedding (Is)

$$Is_{(t+1,a=h+1)} = \ \text{N}Is_{(t+1,a)}$$

$$Is_{(t+1,a\in[h+2;cal])} = \ \left[Is_{(t,a-1)} - sIs_{(t,a-1)}\right] - \text{N}Ic_{(t+1,a)} + \text{N}Is_{(t+1,a)}$$

$-$

$$Is_{(t+1,P_1)} = \ \left[Is^{sg}_{(t,P_1)} - sIs^{sg}_{(t,P_1)} + Is_{(t,cal)} - sIs_{(t,cal)}\right] - \text{N}Ic_{(t+1,P_1)} + \text{N}Is_{(t+1,P_1)}$$

$$Is_{(t+1,P_i\in[P_2;P_4])} = \left[Is^{sg}_{(t,P_i)} - sIs^{sg}_{(t,P_i)} + Is^{ng}_{(t,P_{i-1})} - sIs^{ng}_{(t,P_{i-1})}\right] - \text{N}Ic_{(t+1,P_i)} + \text{N}Is_{(t+1,P_i)}$$

$$Is_{(t+1,P_{5+})} = \left[Is_{(t,P_{5+})} - sIs_{(t,P_{5+})} + Is^{ng}_{(t,P_4)} - sIs^{ng}_{(t,P_4)}\right] - \text{N}Ic_{(t+1,P_{5+})} + \text{N}Is_{(t+1,P_{5+})}$$

High shedding and clinically affected (Ic)

$$Ic_{(t+1,a=h+2)} = \ \text{N}Ic_{(t+1,a)}$$

$$Ic_{(t+1,a\in[h+3;cal])} = \ \left[Ic_{(t,a-1)} - sIc_{(t,a-1)}\right] + \text{N}Ic_{(t+1,a)}$$

$-$

$$Ic_{(t+1,P_1)} = \ \left[Ic^{sg}_{(t,P_1)} - sIc^{sg}_{(t,P_1)} + Ic_{(t,cal)} - sIc_{(t,cal)}\right] + \text{N}Ic_{(t+1,P_1)}$$

$$Ic_{(t+1,P_i\in[P_2;P_4])} = \left[Ic^{sg}_{(t,P_i)} - sIc^{sg}_{(t,P_i)} + Ic^{ng}_{(t,P_{i-1})} - sIc^{ng}_{(t,P_{i-1})}\right] + \text{N}Ic_{(t+1,P_i)}$$

$$Ic_{(t+1,P_{5+})} = \left[Ic_{(t,P_{5+})} - sIc_{(t,P_{5+})} + Ic^{ng}_{(t,P_4)} - sIc^{ng}_{(t,P_4)}\right] + \text{N}Ic_{(t+1,P_{5+})}$$

Equations describing flows

New incoming (and outgoing) flows in each health states are mainly drawn using binomial laws.

Births (bX)

At each time step t, births are calculated with regards to the health state of the dam. These births are then distributed into S and T states

$$bS_{(t)} = \ bS^{R}_{(t)} + bS^{L}_{(t)} + bS^{Is}_{(t)} + bS^{Ic}_{(t)} \qquad \text{et} \qquad bT_{(t)} = \ bT^{L}_{(t)} + bT^{Is}_{(t)} + bT^{Ic}_{(t)},$$

where $bS^{X}_{(t)}$ et $bT^{X}_{(t)}$ ($X \in R, L, Is, Ic$) represent the number of births at time t from cows in health state X:

$$\begin{aligned}
&bS^{R}_{(t)} \sim Bin\left(R_{(t,cal)} + \sum_{i=1}^{i=5}\left[R^{ng}_{(t,P_i)}\right]; 1-\sigma_B\right)\\
&bS^{L}_{(t)} = nbV^{L}_{(t)} - bT^{L}_{(t)}, \quad bT^{L}_{(t)} \sim Bin\left(nbV^{L}_{(t)}; p_L * \varphi_{p_{LIs}}\right)\\
&bS^{Is}_{(t)} = nbV^{Is}_{(t)} - bT^{Is}_{(t)}, \quad bT^{Is}_{(t)} \sim Bin\left(nbV^{Is}_{(t)}; p_{Is} * \varphi_{p_{LIs}}\right)\\
&bS^{Ic}_{(t)} = nbV^{Ic}_{(t)} - bT^{Ic}_{(t)}, \quad bT^{Ic}_{(t)} \sim Bin\left(nbV^{Ic}_{(t)}; p_{Ic} * \varphi_{p_{Ic}}\right)
\end{aligned}$$

In equations above, $nbC^{X}_{(t)}$ is the number of female calves alive born at time t. It is obtained from $nbV^{X}_{(t)}$, the number of female calves born at time t, from cows in the health state X:

$$\begin{aligned}
&nbV^{L}_{(t)} \sim Bin\left(L_{(t,cal)} + T_{(t,cal)} + \sum_{i=1}^{i=5}\left[L^{ng}_{(t,P_i)}\right]; 1-\sigma_B\right)\\
&nbV^{Is}_{(t)} \sim Bin\left(Is_{(t,cal)} + \sum_{i=1}^{i=5}\left[Is^{ng}_{(t,P_i)}\right]; 1-\sigma_B\right)\\
&nbV^{Ic}_{(t)} \sim Bin\left(Ic_{(t,cal)} + \sum_{i=1}^{i=5}\left[Ic^{ng}_{(t,P_i)}\right]; 1-\sigma_B\right)
\end{aligned}$$

We note that at the age of moving in the adult group (cal), all the animals in the health state T enter the health state L ($L_{(t,cal)} = L_{(t,cal)} + T_{(t,cal)}$). φ_{p_x} is a factor, varying from 100% to 0%, of decrease in probability of in utero transmission where x correspond to the dam giving birth in health states L and Is ($\varphi_{p_{LIs}}$) or Ic ($\varphi_{p_{Ic}}$).

Change in age group (X^x)

$$\begin{aligned}
&X_{(t,P_i)} = X^{sg}_{(t,P_i)} + X^{ng}_{(t,P_i)}\\
&X^{ng}_{(t,P_i)} \sim Bin\left(X_{(t,P_i)}; \frac{1}{\tau_{aa}}\right) \quad ; \quad \text{where } X = \{R, L, Is, Ic\},
\end{aligned}$$

and τ_{aa} is the average time spent in each of age group P_1 to P_4.

Exits (sX)

The mortality of calves during the first week of life is applied at birth and defined above in the section concerning births.
From age 1 to cal, mortality and culling rates σ_x are defined as:

- $a \in \{1; 2\} \rightarrow \sigma_x = \sigma_{c1}$
- $a \in [3; w] \rightarrow \sigma_x = \sigma_{c2}$
- $a \in [w+1; cal] \rightarrow \sigma_x = \sigma_{c3}$

Then, exits following death and culling write as:
for $a = 1$:

$$sX_{(t,a)} \sim Bin\left(X_{(t,a)}; \sigma_x\right), \quad \text{where } X = \{S, T\},$$

for $a \in [2; 4]$:

$$\begin{aligned} &sX_{(t,a)} = sale + death, \quad \text{where } X = \{S, T, L\} \\ &sale \sim Bin\left(X_{(t,a)}; \sigma_m\right) \\ &death \sim Bin\left(X_{(t,a)} - sale; \sigma_x\right) \end{aligned}$$

for $a \in [5; cal - 11]$:

$$sX_{(t,a)} \sim Bin\left(X_{(t,a)}; \sigma_x\right) \quad ; \quad X = \{S, R, T, L\}$$

for $a = cal - 10$, we consider management by heifers (safe management, keep all female calves):

$$\begin{aligned} &Cows_{(t)} = \sum_{i=1}^{5} R_{(t,P_i)} + \sum_{i=1}^{5} L_{(t,P_i)} + \sum_{i=1}^{5} Is_{(t,P_i)} + \sum_{i=1}^{5} Ic_{(t,P_i)} \\ &Heifers_{(t)} = \sum_{a=1}^{u} S_{(t,a)} + \sum_{a=u+1}^{cal} R_{(t,a)} + \sum_{a=1}^{cal} T_{(t,a)} + \sum_{a=1}^{cal} L_{(t,a)} + \sum_{a=1}^{cal} Is_{(t,a)} + \sum_{a=1}^{cal} Ic_{(t,a)} \end{aligned}$$

If the number of heifers, $Heifers_{(t)}$, is greater than K_g or that the number of cows, $Cows_{(t)}$, is greater than K_v, we consider the sale of heifers:

$$\begin{aligned} &sX_{(t,a)} = sale + death \quad ; \quad X = \{R, T, L, Is, Ic\} \\ &sale \sim Bin\left(X_{(t,a)}; \exp\left(-\sigma_h.(Cows_{(t)}/K_v)^6\right).((Heifers_{(t)}/K_g)^6)\right) \\ &death \sim Bin\left(X_{(t,a)} - sale; \sigma_x\right) \end{aligned}$$

where K_v is the capacity of the holding in number of cows (see Table 1) and $K_g = \sigma_P * K_v * (cal - h)$ is the capacity of the holding in number of heifers.

Otherwise, we do not consider the sale of heifers:

$$sX_{(t,a)} \sim Bin\left(X_{(t,a)}; \sigma_x\right)$$

for $a \in [cal-9; cal]$:

$$sX_{(t,a)} = Bin\left(X_{(t,a)}; \sigma_x\right)$$

for $a \in [P_1; P_{5+}]$:

$$sX^x_{(t,P_i)} \sim Bin\left(X^x_{(t,P_i)}; \sigma_{Pi}\right), \quad \text{where } X = \{R, L, Is\},$$
$$sIc^x_{(t,P_i)} \sim Bin\left(Ic^x_{(t,P_i)}; \frac{1}{v_{Ic}}\right)$$

where v_{Ic} corresponds to the average time spent in the health state Ic.

New infections (S → T, except for *in-utero* transmission)

We have:

$$\text{N}T_{(t+1,a)} = inf^c_{(t+1,a)} + inf^m_{(t+1,a)} + inf^l_{(t+1,a)} + inf^g_{(t+1,a)}$$

Superscripts correspond to different possible routes of transmission, respectively colostrum (c), milk (m), local environment (l) and global environment (g). New infections by *in-utero* transmission are accounted for through births.

By age, the possible routes of infection are:

$$0 \underbrace{\longrightarrow}_{cmlg} 1 \underbrace{\rightarrow \cdots \rightarrow}_{mlg} \text{weaning} \underbrace{\rightarrow \cdots \rightarrow}_{lg} \text{grazing allowed} \underbrace{\rightarrow \cdots \rightarrow}_{\substack{\text{outdoor: } l \\ \text{indoor: } lg}} \text{limit of susceptibility}$$

Transmission through colostrum

It is consideblackcol that calves drink colostrum from their mothers during the first three days before drinking milk:

$$inf^c_{(t+1,a=1)} = \sum_{1}^{bS^{Is}_{(t)}} \left[Bern\left(1 - exp\left(-\frac{\beta_l \; q^{Is}_c}{\alpha}\right)\right)\right] + \sum_{1}^{bS^{Ic}_{(t)}} \left[Bern\left(1 - exp\left(-\frac{\beta_l \; q^{Ic}_c}{\alpha}\right)\right)\right],$$

with

$$q^{Is}_c \sim Bern(sh_{Is}) \times \left[3 \times b\left(10^5.Beta(8;8) + 1 + 10^3.Beta(1;25)\right)\right] * \varphi Milk_{Is},$$
$$q^{Ic}_c \sim Bern(sh_{Ic}) \times \left[3 \times b\left(10^5.Beta(8;8) + 10^{(3+10.Beta(50;200))}\right)\right] * \varphi Milk_{Ic}.$$

Where $\varphi Milk_x$ is a factor, varying from 100% to 0%, of decrease in quantity of bacteria shed in colostrum and milk by an animal in health state x (here x could be Is or Ic)

Transmission through milk

Regarding the age, we have:

$$a = 1: \quad inf^{m}_{(t+1,a)} \sim Bin\left(bS; 1 - exp\left(-\frac{\beta_l \ q_l \ \frac{4}{7}}{\alpha}\right)\right),$$
$$a \in \{2; w\}: \quad inf^{m}_{(t+1,a)} \sim Bin\left(\left[S_{(t,a-1)} - sS_{(t,a-1)}\right]; \left[1 - exp\Big(-e^{(-\gamma(a-1))}.\frac{\beta_l \ q_l}{\alpha}\Big)\right]\right),$$

with

$$q_l = \frac{7 \times d \times \left(Q^{milk}_{Is(t)} + Q^{milk}_{Ic(t)}\right)}{MilkTot_{(t)}},$$

where

$$Q^{milk}_{Is(t)} = \ 7 \times \varepsilon \times g_{Is} \times \left(\sum_{1}^{nbExcr^{Is}_{(t)}} (10^5.Beta(8;8)) + \sum_{1}^{nbLac^{Is}_{(t)}} (1 + 10^3.Beta(1;25))\right) * \varphi Milk_{Is},$$
$$Q^{milk}_{Ic(t)} = \ 7 \times \varepsilon \times g_{Ic} \times \left(\sum_{1}^{nbExcr^{Ic}_{(t)}} (10^5.Beta(8;8)) + \sum_{1}^{nbLac^{Ic}_{(t)}} 10^{(3+10.Beta(50;200))}\right) * \varphi Milk_{Ic},$$
$$MilkTot_{(t)} = \ 7 \times \varepsilon \times \left(nbLac^{R}_{(t)} + g_L.nbLac^{L}_{(t)} + g_{Is}.nbLac^{Is}_{(t)} + g_{Ic}.nbLac^{Ic}_{(t)}\right).$$

with

$$nbExcr^{Is}_{(t)} \sim Bin\left(nbLac^{Is}_{(t)}; sh_{Is}\right),$$
$$nbExcr^{Ic}_{(t)} \sim Bin\left(nbLac^{Ic}_{(t)}; sh_{Ic}\right),$$
$$nbLac^{R}_{(t)} \sim Bin\left(\sum_{i=1}^{i=5} R_{(t,P_i)}, prop\right),$$
$$nbLac^{L}_{(t)} \sim Bin\left(\sum_{i=1}^{i=5} L_{(t,P_i)}, prop\right),$$
$$nbLac^{Is}_{(t)} \sim Bin\left(\sum_{i=1}^{i=5} Is_{(t,P_i)}, prop\right),$$
$$nbLac^{Ic}_{(t)} \sim Bin\left(\sum_{i=1}^{i=5} Ic_{(t,P_i)}, prop\right).$$

Local transmission (in collective pens, $a \in [1; u]$)

$$inf^{l}_{(t+1,a)} \sim Bin\left(S_{(t,a-1)} - sS_{(t,a-1)}; p^{i}_{inf(t+1,a)}\right),$$

where

during housing period:

$$p^i_{inf(t+1,a)} = 1 - exp\left(-e^{(-\gamma(a-1))}.\frac{\beta_c E^i_{(t+1)}}{\alpha N^i_{(t+1)}}\right), \text{ and}$$

during grazing period, regarding the age,

$$a \in [1;26]: \quad p^i_{inf(t+1,a)} = 1 - exp\left(-e^{(-\gamma(a-1))}.\frac{\beta_c E^i_{(t+1)}}{\alpha N^i_{(t+1)}}\right),$$

$$a \in [27;u]: \quad p^i_{inf(t+1,a)} = 1 - exp\left(-e^{(-\gamma(a-1))}.\frac{\beta_o E^i_{(t+1)}}{\alpha N^i_{(t+1)}}\right).$$

$N^i_{(t+1)}$ is the total number of animals in environment i, across all health states, and E^i represents the quantity of bacteria in the environment, with i corresponding to the specific area.

Global transmission

This occurs up to the age allowing to go to the pasture (26 weeks - 6 months) during the grazing period, and up to the age limit for sensitivity (u) during the housing period:

$$inf^g_{(t+1,a)} \sim Bin\left(S_{(t,a)} - sS_{(t,a)}; p^g_{inf(t+1,a)}\right),$$

where

$$p^g_{inf(t+1,a)} = 1 - exp\left(-exp[-h(a-1)].\frac{\beta_g E^g_{(t+1)}}{\alpha N^g_{(t+1)}}\right), \quad \text{with} \quad E^g_{(t+1)} = \sum_{i=1}^{i=5} E^{\text{INT}i}_{(t+1)}$$

$E^{\text{INT}i}$ represents the quantity of bacteria in the environment where INTi corresponds to a specific area (see the section below about the dynamics of bacteria in the environments).

New latently infected (T → L)

For $a < cal$:

$$\text{N}L_{(t+1,a\in[2;cal-1])} \sim Bin\left(T_{(t,a)} - sT_{(t,a)}; \frac{1}{\upsilon_T}\right).$$

After age cal, there are no more animals in T state:

$$\text{N}L_{(t+1,P_1)} = T_{(t,cal)} - sT_{(t,cal)}.$$

New subclinically infected (L → Is)

For heifers:

$$\textsc{n}Is_{(t+1,a\in[h+1;cal])} \sim Bin\left(L_{(t,a)} - sL_{(t,a)}; \frac{1}{v_L}\right).$$

For cows:

$$\textsc{n}Is_{(t+1,P_i)} \sim Bin\left(n; \frac{1}{v_L}\right),$$

with, regarding the age,

$$\begin{aligned}
P_1 \rightarrow n &= \left[L^{sg}_{(t,P_1)} - sL^{sg}_{(t,P_1)} + L_{(t,cal)} - sL_{(t,cal)} + T_{(t,cal)} - sT_{(t,cal)}\right],\\
\{P_2;P_4\} \rightarrow n &= \left[L^{sg}_{(t,P_i)} - sL^{sg}_{(t,P_i)} + L^{ng}_{(t,P_{i-1})} - sL^{ng}_{(t,P_{i-1})}\right],\\
P_{5+} \rightarrow n &= \left[L_{(t,P_{5+})} - sL_{(t,P_{5+})} + L^{ng}_{(t,P_4)} - sL^{ng}_{(t,P_4)}\right].
\end{aligned}$$

New clinically infected (Is → Ic)

For heifers:

$$\textsc{n}Ic_{(t+1,a\in[h+1;cal])} \sim Bin\left(Is_{(t,a)} - sIs_{(t,a)}; \frac{1}{v_{Is}}\right).$$

For cows:

$$\textsc{n}Ic_{(t+1,P_i)} \sim Bin\left(n; \frac{1}{v_{Is}}\right),$$

with

$$\begin{aligned}
P_1 \rightarrow n &= \left[Is^{sg}_{(t,P_1)} - sIs^{sg}_{(t,P_1)} + Is_{(t,cal)} - sIs_{(t,cal)}\right],\\
\{P_2;P_4\} \rightarrow n &= \left[Is^{sg}_{(t,P_i)} - sIs^{sg}_{(t,P_i)} + Is^{ng}_{(t,P_{i-1})} - sIs^{ng}_{(t,P_{i-1})}\right],\\
P_{5+} \rightarrow n &= \left[Is_{(t,P_{5+})} - sIs_{(t,P_{5+})} + Is^{ng}_{(t,P_4)} - sIs^{ng}_{(t,P_4)}\right].
\end{aligned}$$

New resistant (S → R)

At age u, the transition from compartment S to compartment R is done in a deterministic way.

Dynamics of bacteria in the environments (E)

The composition of the environments according to the season is the following:

$$\text{Grazing}: a = \overbrace{1\cdots\cdots\cdots w}^{\textsc{int}1}\overbrace{\cdots\cdots\cdots 26}^{\textsc{int}2}\overbrace{\cdots\cdots\cdots\cdots\cdots\cdots\cdots\cdots h}^{\textsc{ext}1+\textsc{ext}2}\overbrace{\cdots\cdots\cdots\cdots cal}^{\textsc{ext}3}$$

$$\text{Housing} : a = \overbrace{1 \cdots\cdots\cdots w}^{\text{INT1}} \overbrace{\cdots\cdots\cdots\cdots\cdots\cdots\cdots y}^{\text{INT2}} \overbrace{\cdots\cdots\cdots\cdots\cdots h}^{\text{INT3}} \overbrace{\cdots\cdots\cdots\cdots\cdots cal}^{\text{INT4}}$$

Dynamics of bacteria in the environments (E) are defined below:

$E^{\text{INT1}}_{(t+1)} = E^{\text{INT1}}_{(t)}.(1 - \mu_g^{int}) + Q^{\text{TNS}}_{(t)}$. If pens are empty, it becomes $E^{\text{INT1}}_{(t+1)} = E^{\text{INT1}}_{(t+1)}.(1 - \mu_{cp})$, where Q represents the quantity of bacteria shed.

During grazing period, we have:

$$\begin{aligned}
&E^{\text{INT2}}_{(t+1)} = E^{\text{INT2}}_{(t)}.(1 - \mu_g^{int}) + Q^{\text{TS1}}_{(t)} \\
&\qquad If \quad \sum_{a=w+1}^{a=26} SR_{t,a} + \sum_{a=w+1}^{a=26} T_{t,a} + \sum_{a=w+1}^{a=26} L_{t,a} == 0 \quad then \quad E^{\text{INT2}}_{(t+1)} = E^{\text{INT2}}_{(t+1)}.(1 - \mu_{cp}) \\
&E^{\text{INT3}}_{(t+1)} = E^{\text{INT3}}_{(t)}.(1 - \mu_g^{int}) \\
&E^{\text{INT4}}_{(t+1)} = E^{\text{INT4}}_{(t)}.(1 - \mu_g^{int}) \\
&E^{\text{INT5}}_{(t+1)} = E^{\text{INT5}}_{(t)}.(1 - \mu_g^{int}) \\
&E^{\text{EXT1}}_{(t+1)} = E^{\text{EXT1}}_{(t)}.(1 - \mu_g^{ext}) + Q^{\text{TS2}}_{(t)} \\
&E^{\text{EXT2}}_{(t+1)} = E^{\text{EXT2}}_{(t)}.(1 - \mu_g^{ext}) + Q^{\text{TY}}_{(t)} \\
&E^{\text{EXT3}}_{(t+1)} = E^{\text{EXT3}}_{(t)}.(1 - \mu_g^{ext}) + Q^{\text{TH}}_{(t)} + Q^{\text{ISH}}_{(t)} + Q^{\text{ICH}}_{(t)}.
\end{aligned}$$

During housing period, we have:

$$\begin{aligned}
&E^{\text{INT2}}_{(t+1)} = E^{\text{INT2}}_{(t)}.(1 - \mu_g^{int}) + Q^{\text{TS1}}_{(t)} + Q^{\text{TS2}}_{(t)} \\
&\qquad If \quad \sum_{a=w+1}^{a=52} SR_{t,a} + \sum_{a=w+1}^{a=52} T_{t,a} + \sum_{a=w+1}^{a=52} L_{t,a} == 0 \quad then \quad E^{\text{INT2}}_{(t+1)} = E^{\text{INT2}}_{(t+1)}.(1 - \mu_{cp}) \\
&E^{\text{INT3}}_{(t+1)} = E^{\text{INT3}}_{(t)}.(1 - \mu_g^{int}) + Q^{\text{TY}}_{(t)} \\
&E^{\text{INT4}}_{(t+1)} = E^{\text{INT4}}_{(t)}.(1 - \mu_g^{int}) + Q^{\text{TH}}_{(t)} + Q^{\text{ISH}}_{(t)} + Q^{\text{ICH}}_{(t)} \\
&E^{\text{INT5}}_{(t+1)} = E^{\text{INT5}}_{(t)}.(1 - \mu_g^{int}) + Q^{\text{IS}}_{(t)} + Q^{\text{IC}}_{(t)} \\
&E^{\text{EXT1}}_{(t+1)} = 0, \quad E^{\text{EXT2}}_{(t+1)} = 0, \quad E^{\text{EXT3}}_{(t+1)} = 0.
\end{aligned}$$

Shed quantities of bacteria are defined, regarding the health states and the age, by:

unweaned calves T:

$$Q^{\text{TNS}}_{(t)} = \sum_{a=1}^{a=w} \left[7.f_1.10^6. \sum^{T_{(t,a)}} Beta(8.8; 19) \right] * \varphi faeces_T,$$

weaned calves T, without access to grazing:

$$Q^{\text{TS1}}_{(t)} = \sum_{a=w+1}^{a=26} \left[7.f_2.10^6. \sum^{T_{(t,a)}} Beta(8.8; 19) \right] * \varphi faeces_T,$$

weaned calves T, with access to grazing:

$$Q^{\text{Ts2}}_{(t)} = \sum_{a=27}^{a=y} \left[7.f_2.10^6. \sum^{T_{(t,a)}} Beta(8.8;19) \right] * \varphi faeces_T,$$

young heifers T:

$$Q^{\text{TY}}_{(t)} = \sum_{a=y+1}^{a=h} \left[7.f_Y.10^6. \sum^{T_{(t,a)}} Beta(8.8;19) \right] * \varphi faeces_T,$$

heifers T:

$$Q^{\text{TH}}_{(t)} = \sum_{a=h+1}^{a=cal} \left[7.f_A.10^6. \sum^{T_{(t,a)}} Beta(8.8;19) \right] * \varphi faeces_T,$$

heifers Is:

$$\text{if} \sum_{a=h+1}^{a=cal} \left(Is_{(t,a)} \right) > 0 \ : \quad Q^{\text{IsH}}_{(t)} = \sum_{a=h+1}^{a=cal} \left[7.f_A.10^{(4+10\times\sum^{Is_{(t,a)}} Beta(2.65;17))} \right] * \varphi faeces_{Is}$$

$$\text{else} \quad Q^{\text{IsH}}_{(t)} = 0,$$

cows Is:

$$\text{if} \sum_{i=1}^{i=5} \left(Is_{(t,P_i)} \right) > 0 \ : \quad Q^{\text{Is}}_{(t)} = \sum_{i=1}^{i=5} \left[7.f_A.10^{(4+10\times\sum^{Is_{(t,P_i)}} Beta(2.65;17))} \right] * \varphi faeces_{Is}$$

$$\text{else} \quad Q^{\text{Is}}_{(t)} = 0,$$

heifers Ic:

$$\text{if} \sum_{a=h+1}^{a=cal} \left(Ic_{(t,a)} \right) > 0 \ : \quad Q^{\text{IcH}}_{(t)} = \sum_{a=h+1}^{a=cal} \left[7.f_A.10^{(8+10\times\sum^{Ic_{(t,a)}} Beta(2;17))} \right] * \varphi faeces_{Ic}$$

$$\text{else} \quad Q^{\text{IcH}}_{(t)} = 0,$$

cows Ic:

$$\text{if} \sum_{i=1}^{i=5} \left(Ic_{(t,P_i)} \right) > 0 \ : \quad Q^{\text{Ic}}_{(t)} = \sum_{i=1}^{i=5} \left[7.f_A.10^{(8+10\times\sum^{Ic_{(t,P_i)}} Beta(2;17))} \right] * \varphi faeces_{Ic}$$

$$\text{else} \quad Q^{\text{Ic}}_{(t)} = 0.$$

$\varphi faeces_x$, in the equation above is a factor of decrease in quantity of bacteria shed by an animal in health state x. x could be T, Is or Ic.

B Parameters ralated to population dynamics

Table 1: Parameters for management and population dynamics used in the *Mycobacterium avium subsp. paratuberculosis* (*Map*) infection dynamics model within a structublackcol dairy herd (reproduced from [2], Table 1).

Notation	Value	Definition	Source
σ_B	0.07	Mortality rate of calves at birth	$*$, [17]
σ_m	0.206	Exit rate of male calves, weeks 2 to 4 (per week)	
σ_{c1}	0.015	Death rate of female calves, weeks 1 and 2 (per week)	[17]
σ_{c2}	0.0035	Death rate of female calves, weeks 3 to weaning (per week)	[9]
σ_{c3}	0.00019	Death rate of heifers from weaning to entry in adult group (per week)	†
σ_h	0.011	Sale rate of bblackcol heifers 10 weeks before 1st calving	†
σ_{Ai}	0.27,0.25,0.31, 0.31,0.62	Yearly culling rate of cows in adult group i: 1, 2, 3, 4 and above 5 respectively (%)	$*$, [1]
w	10	Weaning age (weeks)	[12]
y	52	Age when entering the young heifer group (weeks)	
h	91	Age when entering the heifer group (weeks)	$*$
cal	130	Age when entering the adult group (weeks)	$*$, †
τ_{aa}	56.3	Mean time spent in adult age groups 1 to 4 (weeks)	$*$, †
b	5	Quantity of colostrum fed to calves (L/day for 3 days)	†
d	7	Quantity of milk fed to calves after 3 days (L/day/calf)	†
$prop$	0.85	Proportion of lactating cows	$*$
ε	25	Quantity of milk or colostrum produced (L/day/cow)	$*$
f_1	0.5	Quantity of feaces produced by a non-weaned calf (kg/day)	†
f_2	5.5	Quantity of feaces produced by a weaned calf (kg/day)	†
f_Y	10	Quantity of feaces produced by a heifer (kg/day)	†
f_A	30	Quantity of feaces produced by a cow (kg/day)	†
$Graz$	$[14-26]$	Grazing period (1 being the first week of the year)	†
K_c	110	Number of cows above which the heifer selling rate increases	

$*$ Agricultural statistics.
† Based on expert opinion.

C Parameters related to infection dynamics

Table 2: Parameters for infection and transmission used in the *Mycobacterium avium subsp. paratuberculosis* (*Map*) infection dynamics model within a structublackcol dairy herd (reproduced from [2] - Table 2).

Notation	Value	Definition	Source
p_X		Probability of *in utero* transmission for cow in health state X	[4, 26]
	$p_L = 0.149$	X = latently infected (L)	
	$p_{Is} = 0.149$	X = subclinically infected (Is)	
	$p_{Ic} = 0.65$	X = clinically affected (Ic)	
u	52	Maximal age in the susceptible compartment (weeks)	[6, 23]
γ	0.1	Susceptibility follows an exponential decrease : $exp(-\gamma(age-1))$	[27]
v_X		Mean time spent in health state X (weeks)	
	$v_T = 25$	X = transiently infectious (T)	[21]
	$v_L = 52$	X = latently infected (L)	[16, 14]
	$v_{Is} = 104$	X = subclinically infected (Is)	[13]
	$v_{Ic} = 26$	X = clinically affected (Ic)	†
sh_X		Probability of shedding in colostrum or milk for a cow in health state X	[20, 19]
	$sh_L = 0$	X = latently infected (L)	
	$sh_{Is} = 0.4$	X = subclinically infected (Is)	
	$sh_{Ic} = 0.9$	X = clinically affected (Ic)	
α	10^6	*Map* infectious dose	[3]
β_l	$5 \times 10^{-4} \times 7$	Transmission rate if ingestion of an infectious dose (per week)	‡
β_c	$5 \times 10^{-5} \times 7$	Transmission rate if one infectious dose is present in the local environment (per week)	[21]
β_g	$9.5 \times 10^{-7} \times 7$	Transmission rate if one infectious dose is present in the global environment (per week)	[21]
β_o	$5 \times 10^{-6} \times 7$	Transmission rate if one infectious dose is present in the pasture (per week)	‡
g_X		Decrease in milk production for cattle in health state X (per week)	[15]
	$g_{Is} = 1 - 0.11$	X = subclinically infected (Is)	
	$g_{Ic} = 1 - 0.25$	X = clinically affected (Ic)	
μ_k		Removal rate of Map from environment k	[7, 24]
	$\mu_g^{int} = 0.4$	all the environments (per week)	
	$\mu_g^{ext} = 1/14$	all the environments (per week)	
	$\mu_{cp} = 0.17$	collective pens (when empty)	

† Expert opinions.

‡ Parameters' values are assumed.

Table 3: Summary of published data and assumed distributions of the quantities of *Mycobacterium avium* subsp. *paratuberculosis* (*Map*) shed, depending on the health state (X) and the route of transmission (r) in the *Map* infection dynamics model within a structublackcol dairy herd (reproduced from [2] - Table 3).

**Route of transmission (r)**	**Health state (X)**	**Literature**				**Assumed distributions**
		**Minimal value**	**Maximal value**	**Mean value**	**Source**	
Map direct shedding in milk and colostrum (Map/L)	Subclinically infected	2.2×10^4	8.8×10^4	5×10^4	[20]	$10^5 \times Beta(8;8)$
	Clinically affected	-	-	5×10^4	[5]	$10^5 \times Beta(8;8)$
Map indirect shedding in milk and colostrum (faecal contamination) (Map/L)	Subclinically infected	0	2×10^{10}	40	[10, 22]	$1 + 10^3 \times Beta(1;25)$
	Clinically affected	700	2×10^{10}	14×10^4	[10, 22]	$10^{(3+10\times Beta(50;200))}$
Map shedding in faeces (Map/kg)	Transiently infectious	6×10^4	6.3×10^5	3×10^5	[21]	$10^6 \times Beta(8.8;19)$
	Subclinically infected	10^4	10^{15}	2.6×10^6	[18]	$10^{(4+10\times Beta(2.65;17))}$
	Clinically affected	10^8	10^{15}	10^{10}	[25, 8]	$10^{(8+10\times Beta(2;17))}$

Bibliography

[1] Beaudeau, F. (1999). *Review of culling and replacement practices in dairy cattle herds, Master Thesis. Institut Supérieur des Productions Animales*. PhD thesis, Rennes, France.

[2] Beaunée, G., Vergu, E., and Ezanno, P. (2015). Modelling of paratuberculosis spread between dairy cattle farms at a regional scale. *Veterinary Research*, 46:1–13.

[3] Begg, D. J. and Whittington, R. J. (2008). Experimental animal infection models for Johne's disease, an infectious enteropathy caused by Mycobacterium avium subsp. paratuberculosis. *The Veterinary Journal*, 176(2):129–145.

[4] Benedictus, A., Mitchell, R. M., Linde-Widmann, M., Sweeney, R., Fyock, T., Schukken, Y. H., and Whitlock, R. H. (2008). Transmission parameters of Mycobacterium avium subspecies paratuberculosis infections in a dairy herd going through a control program. *Preventive Veterinary Medicine*, 83(3-4):215–227.

[5] Giese, S. B. and Ahrens, P. (2000). Detection of Mycobacterium avium subsp paratuberculosis in milk from clinically affected cows by PCR and culture. *Veterinary Microbiology*, 77(3-4):291–297.

[6] Hagan, W. A. (1938). Age as a Factor in Susceptibility to Johne's Disease. *Cornell Veterinarian*, 28:34–40.

[7] Jørgensen, J. B. (1977). Survival of Mycobacterium paratuberculosis in slurry. *Nordisk Veterinaer Medicin*, 29(6):267–270.

[8] Jørgensen, J. B. (1982). An improved medium for culture of Mycobacterium paratuberculosis from bovine faeces. *Acta veterinaria Scandinavica*, 23(3):325–335.

[9] Jégou, V., Porhiel, J., and Brunschwig, P. (2006). Risk management factors affecting mortality among dairy calves herds in 80 herds in Brittany. In *Proc. Journées Bovines Nantaises*, Nantes.

[10] Magnusson, M., Christiansson, A., Svensson, B., and Kolstrup, C. (2006). Effect of Different Premilking Manual Teat-Cleaning Methods on Bacterial Spores in Milk. *Journal of Dairy Science*, 89(10):3866–3875.

[11] Marcé, C., Ezanno, P., Seegers, H., Pfeiffer, D. U., and Fourichon, C. (2011). Predicting fadeout versus persistence of paratuberculosis in a dairy cattle herd for management and control purposes: a modelling study. *Veterinary Research*, 42(36).

[12] Marcé, C., Guatteo, R., Bareille, N., and Fourichon, C. (2010). Dairy calf housing systems across Europe and risk for calf infectious diseases. *animal*, 4(09):1588–1596.

[13] Matthews, H. T. (1947). On Johne's disease. *The Veterinary Record*, 59(31):397–401.

[14] Nielsen, S. S. (2008). Transitions in diagnostic tests used for detection of Mycobacterium avium subsp. paratuberculosis infections in cattle. *Veterinary Microbiology*, 132(3-4):274–282.

[15] Nielsen, S. S., Enevoldsen, C., and Toft, N. (2006). Milk production losses associated with bovine paratuberculosis diagnosed from repeated testing. In *11th international Symposia of Veterinary Epidemiology and Economics*, Cairns.

[16] Nielsen, S. S. and Ersbøll, A. K. (2006). Age at Occurrence of Mycobacterium avium Subspecies paratuberculosis in Naturally Infected Dairy Cows. *Journal of Dairy Science*, 89(12):4557–4566.

[17] Rio, O. (1999). *Frequency and risks of mortality and health disorders of calves in dairy cattle herds* . PhD thesis, Nantes.

[18] Rossiter, C. A. and Burhans, W. S. (1996). Farm-specific approach to paratuberculosis (Johne's disease) control. *The Veterinary clinics of North America. Food animal practice*, 12(2):383–415.

[19] Streeter, R. N., Hoffsis, G. F., Bechnielsen, S., Shulaw, W. P., and Rings, M. (1995). Isolation of Mycobacterium-Paratuberculosis From Colostrum and Milk of Subclinically Infected Cows. *American Journal of Veterinary Research*, 56(10):1322–1324.

[20] Sweeney, R. W., Whitlock, R. H., and Rosenberger, A. E. (1992). Mycobacterium paratuberculosis isolated from fetuses of infected cows not manifesting signs of the disease. *American Journal of Veterinary Research*, 53(4):477–480.

[21] van Roermund, H. J. W., Bakker, D., Willemsen, P. T. J., and de Jong, M. C. M. (2007). Horizontal transmission of Mycobacterium avium subsp. paratuberculosis in cattle in an experimental setting: Calves can transmit the infection to other calves. *Veterinary Microbiology*, 122(3-4):270–279.

[22] Vissers, M. M. M., Driehuis, F., Te Giffel, M. C., De Jong, P., and Lankveld, J. M. G. (2006). Improving Farm Management by Modeling the Contamination of Farm Tank Milk with Butyric Acid Bacteria. *Journal of Dairy Science*, 89(3):850–858.

[23] Whitlock, R. H. and Buergelt, C. (1996). Preclinical and clinical manifestations of paratuberculosis (including pathology). *Veterinary Clinics of North America-Food Animal Practice*, 12(2):345–356.

[24] Whittington, R. J., Marshall, D. J., Nicholls, P. J., Marsh, I. B., and Reddacliff, L. A. (2004). Survival and dormancy of Mycobacterium avium subsp. paratuberculosis in the environment. *Applied and Environmental Microbiology*, 70(5):2989–3004.

[25] Whittington, R. J., Reddacliff, L. A., Marsh, I., McAllister, S., and Saunders, V. (2000). Temporal patterns and quantification of excretion of Mycobacterium avium subsp paratuberculosis in sheep with Johne's disease. *Australian Veterinary Journal*, 78(1):34–37.

[26] Whittington, R. J. and Windsor, P. A. (2009). In utero infection of cattle with Mycobacterium avium subsp. paratuberculosis: A critical review and meta-analysis. *The Veterinary Journal*, 179(1):60–69.

[27] Windsor, P. A. and Whittington, R. J. (2010). Evidence for age susceptibility of cattle to Johne's disease. *The Veterinary Journal*, 184(1):37–44.
